# The Predictive Role of Indices Indirectly Indicating Hyperlipidemia and Inflammation in Ascending Aortic Aneurysm

**DOI:** 10.7759/cureus.68531

**Published:** 2024-09-03

**Authors:** Hasan Sari, Yunus E Yavuz, Muhammed F Kaleli, Serhat Kesriklioglu, Yakup Alsancak

**Affiliations:** 1 Cardiology, Mut Statate Hospital, Mersin, TUR; 2 Cardiology, Siirt State Hospital, Siirt, TUR; 3 Cardiology, Necmettin Erbakan University Faculty of Medicine, Konya, TUR

**Keywords:** prognostic indicators, pan-immune-inflammation value, atherogenic index of plasma, inflammation, hyperlipidemia, ascending aortic aneurysm

## Abstract

Introduction

Ascending aortic aneurysm (AAA) is an aortic disease that can progress with serious complications. The roles of hyperlipidemia and inflammation in its etiology are controversial. In this study, we aimed to investigate the predictive value of indices that can indirectly reflect hyperlipidemia and inflammation in patients with AAA.

Methods

This prospective study included 146 patients diagnosed with AAA and 88 controls. Demographic data, clinical history and laboratory results, including the atherogenic index of plasma (AIP), atherogenic coefficient (AC), pan-immune-inflammation value (PIV), and systemic immune-inflammation index (SII), were collected. Statistical analyses were performed to evaluate the relationships between these indices and the aortic diameter.

Results

Patients with AAA presented significantly higher PIV levels than controls did (p>0.05). However, no significant differences were observed in the AIP, AC, or SII between the patient and control groups. Furthermore, no significant correlation was found between the aortic diameter and the studied indices (p<0.05). PIV was the only significant parameter with a p-value of 0.026 (AUC: 0.587) according to the ROC analysis.

Conclusion

These findings suggest that while hyperlipidemia, as measured by the AIP and AC, may not play a direct role in AAA progression, inflammation indicated by PIV could be an important factor. Especially in resource-limited settings, these useful indicators can improve AAA management before the disease progresses to an advanced stage.

## Introduction

Aortic aneurysms represent a significant health concern because of their potential for catastrophic rupture and subsequent mortality [1,2]. These vascular dilations predominantly affect the aorta, the body's principal artery, and can arise in various regions, with the ascending aorta being particularly vulnerable. The ascending aorta is of particular interest in this study because it is uniquely susceptible to hemodynamic forces that differ from those experienced by other parts of the aorta, such as the abdominal aorta. These forces include the high pulsatile flow and the direct impact of left ventricular ejection, which may contribute to distinct pathological mechanisms in the formation and progression of aneurysms in this region. The clinical implications of ascending aortic aneurysms (AAA) are profound due to their silent nature and the high mortality associated with rupture or dissection. Unlike abdominal aortic aneurysms, which are often associated with atherosclerosis, ascending aortic aneurysms may develop due to different pathophysiological mechanisms, including genetic predispositions, connective tissue disorders, and inflammatory processes [3]. These differences necessitate a distinct approach in clinical practice, where surveillance and management strategies are tailored to the specific risks associated with aneurysms of the ascending aorta.

The pathogenesis of AAA is multifactorial, with both hyperlipidemia and inflammation playing crucial roles [4]. Elevated lipid levels are traditionally viewed as risk factors for cardiovascular diseases, including aneurysms, due to their role in atherosclerosis [5]. The role of hyperlipidemia in the development and progression of aortic aneurysms, particularly in the ascending aorta, has been the subject of much debate. Some studies suggest that elevated lipid levels, particularly LDL cholesterol, contribute to aneurysm formation through mechanisms similar to those involved in atherosclerosis [6]. However, other studies have found no significant association between lipid levels and aortic aneurysm risk, or even suggest a protective role in certain contexts [7]. For instance, a meta-analysis by Cheng et al. (2019) demonstrated that while hyperlipidemia is a well-established risk factor for cardiovascular diseases, its role in aortic aneurysm progression remains unclear, with some studies indicating no significant effect of lipid-lowering therapies on aneurysm growth or rupture rates [5]. These conflicting findings highlight the need for further research to elucidate the specific relationship between lipid metabolism and aortic aneurysm pathology, particularly in the ascending aorta. The atherogenic index of plasma (AIP) and atherogenic coefficient (AC) serve as an important marker for lipid metabolism abnormalities and can indicate the presence of atherosclerotic risk factors, which may contribute to the development and progression of AAA [8-10].

Inflammation plays a critical role in the pathogenesis of aortic aneurysms [11]. The inflammatory response contributes to the degradation of the extracellular matrix, smooth muscle cell apoptosis, and other pathological changes within the aortic wall. Inflammatory markers such as C-reactive protein and various cytokines have been implicated in aneurysm development, highlighting the importance of inflammation in this vascular disease. Studies have shown that increased levels of these markers correlate with the presence and expansion of aortic aneurysms, suggesting that inflammation is a key driver of aneurysm pathology [12]. The pan-immune-inflammation value (PIV) has emerged as a significant biomarker in cardiology, especially in patients with cardiovascular conditions such as heart failure and hypertension [13-15]. Elevated PIV levels are linked to adverse outcomes, including increased cardiovascular mortality, acute kidney injury, and other severe complications.

The AIP and PIV were selected for this study because they are emerging markers that reflect lipid metabolism and systemic inflammation, respectively. These indices provide a broader understanding of the underlying factors in aortic aneurysm development. By focusing on AIP and PIV, this study aims to explore their potential as new indicators for assessing the risk and progression of ascending aortic aneurysms, offering insights that other traditional markers may not reveal. Although many studies have shown the relationships of the abdominal aorta with hyperlipidemia and inflammation, to the best of our knowledge, studies on the ascending aorta are limited. This study aimed to investigate the role of hyperlipidemia, as indicated by the AIP, and inflammation, as indicated by PIV, in the context of ascending aortic dilatation. By comparing these biomarkers across different aortic diameter ranges, we aimed to elucidate their contributions to the development and progression of AAA.

## Materials and methods

This study was designed to investigate the predictive value of indirect parameters reflecting hyperlipidemia and inflammation in the context of ascending aortic aneurysms. We conducted a prospective study that included 146 patients diagnosed with AAA and 88 controls between May 2022 and February 2023. We received written consent from all patients. Our University's Ethics Committee received ethical approval on 01.04.2022 with reference number 3714. The study was carried out in accordance with the 2013 Declaration of Helsinki and Good Clinical Practices directive.

Study population

The study cohort consisted of patients with AAA diagnosed through imaging studies and confirmed by aortic diameter measurements. The control group was randomly selected from patients whose ascending aortic diameter was less than 40 mm. In 2014, the ESC aortic diseases guideline accepted that the ascending aorta diameter was below 40 mm in normal individuals, and therefore, those with an aortic diameter of 40 mm were included in the control group. Aortic diameter was initially measured by echocardiography (EPIQ 7 digital ultrasound scanner (Philips Medical System, USA)) at the time of diagnosis, followed by more precise measurements using contrast-enhanced CT of the aorta. Aneurysm growth was analysed using repeated measurements at the sinuses of Valsalva and tubular ascending aorta. The exclusion criteria included heart failure and its symptoms, history of acute coronary syndrome, atherosclerotic heart disease, atherosclerotic cerebrovascular disease, recent major surgery, defined as any surgery requiring general anesthesia within the last six months (such as thoracic, abdominal, or hip surgery), malignancy, and severe renal or hepatic dysfunction. Participants with bicuspid aortic valves or previous aortic repairs were excluded from this study.

Data collection

Demographic data, clinical history, and laboratory results were collected for all participants. The primary parameters assessed included the atherogenic index of plasma and the atherogenic coefficient as indicators of hyperlipidemia and the pan-immune-inflammation value and systemic immune-inflammation index as markers of inflammation. These values were calculated via standard biochemical methods and existing formulas. The definitions of the systemic immune-inflammation index (SII), PIV, AIP, and AC are as follows: SII = platelet × neutrophil/lymphocyte counts (10^9^/L); PIV = platelet × neutrophil × monocyte/lymphocyte counts (10^9^/L); AIP = log10(triglycerides/high-density lipoprotein cholesterol); AC (atherogenic coefficient) = (total cholesterol - high-density lipoprotein cholesterol)/high-density lipoprotein cholesterol. AIP, AC, PIV, and SII were chosen due to their emerging relevance in cardiovascular risk assessment, offering a more comprehensive view of lipid metabolism and systemic inflammation compared to traditional biomarkers.

Statistical analysis of data

Data entry and statistical analysis were performed via the SPSS for Windows version 18.0 (SPSS Inc. Chicago, IL, USA) package. The conformity of the data to a normal distribution was examined via visual (histogram and probability graphs) and analytical methods (Kolmogorov, Smirnov). Median (1st quarter‒3rd quarter) values were used in the evaluation of numerical data; frequency distributions and percentages were used to summarize categorical data. The Mann‒Whitney U test and Kruskal‒Wallis H test were used to compare categorical data with numerical data that were not normally distributed. The correlations of numerical variables that were not normally distributed were analyzed with the Spearman correlation coefficient. The chi-square test was used to compare categorical data. The diagnostic decision-making properties of the calculated indices for predicting AAAs were examined with receiver operating characteristic (ROC) curves. p<0.05 was considered statistically significant.

## Results

The study included 146 patients with AAA and 88 controls. The proportion of male patients with AAAs was significantly greater than that of the control group (p=0.002 OR: 1.416 [%95CI: 1.114-1.799]). The presence of hypertension, chronic kidney disease and cerebrovascular disease was significantly greater in the patient group than in the control group (p<0.05). The age, left ventricular end-diastolic diameter (LVEDD), left ventricular end-systolic diameter (LVESD), left atrium (LA) diameter, PAP, urea, potassium, monocyte values and PIV index of patients with AAA were significantly greater than those of the control group (p<0.05). The distribution range of ejection fraction (EF) was wider in the patient group than in the control group (p<0.001) (Table 1).

**Table 1:** Comparison of demographics, disease characteristics, laboratory parameters and indices between the patient and control groups *Chi-square test for comparison of categorical variables; **Mann-Whitney U test for comparison of continuous variables HR: Heart Rate; EF: Ejection Fraction; LVEDD: Left Ventricular End-Diastolic Diameter; LVESD: Left Ventricular End-Systolic Diameter; LA: Left Atrium; PAB: Pulmonary Artery Blood Pressure; HgB: Hemoglobin; WBC: White Blood Cells; PLT: Platelet Count; NEU: Neutrophil; AIP: Atherogenic Index of Plasma; CRI-I: Castelli Risk Index-I; AC: Atherogenic Coefficient; PIV: Pan-immune-Inflammation Value; SII: Systemic Immune-Inflammation Index

	Patient Group (n=146)	Control Group (n=88)	p
	n (%)	n (%)	
Gender			
Female	45 (30.8)	45 (51.1)	0.002*
Male	101 (69.2)	43 (48.9)	
Chronic disease types			
Diabetes mellitus	34 (23.3)	21 (23.9)	0.920*
Hypertension	108 (74.0)	43 (48.9)	<0.001*
Chronic kidney disease	21 (16.4)	1 (1.1)	<0.001*
Heart failure	17 (11.7)	-	-
Cerebrovascular disease	21 (14.4)	1 (1.1)	0.001*
	Median (1-3 Quartiles)	Median (1-3 Quartiles)	
Age	69.00 (57.75-75.25)	58.50 (50.25-65.00)	<0.001**
HR	75.00 (69.00-80.00)	71.00 (65.25-79.75)	0.083**
LVEDD	48.00 (46.00-51.25)	47.00 (43.00-48.00)	<0.001**
LVESD	29.00 (26.00-33.00)	27.00 (25.00-30.00)	<0.001**
LA diameter	38.00 (35.00-42.00)	35.00 (32.00-38.00)	<0.001**
PAB (mmHg)	30.00 (28.00-35.00)	29.00 (25.25-30.00)	<0.001**
Urea	36.55 (29.76-46.17)	29.80 (23.00-36.75)	<0.001**
Creatinine	1.00 (0.81-1.17)	0.87 (0.76-1.00)	0.001**
Sodium	139.00 (138.00-141.00)	139.00 (138.00-141.00)	0.756**
Potassium	4.44 (4.19-4.78)	4.29 (4.02-4.47)	<0.001**
HgB	14.00 (12.70-15.22)	13.65 (12.42-15.30)	0.657**
WBC	7.77 (6.45-9.25)	8.04 (6.52-9.07)	0.867**
PLT	237.50 (191.00-292.00)	248.50 (203.50-310.00)	0.304**
NEU	4.60 (3.69-5.97)	4.60 (3.81-5.80)	0.739**
Lymphocyte	2.15 (1.68-2.59)	2.29 (1.71-2.80)	0.163**
Monocyte	0.60 (0.45-0.74)	0.50 (0.37-0.62)	<0.001**
Triglyceride	144.55 (101.17-213.02)	159.00 (114.00-202.50)	0.391**
LDL	108.05 (81.01-134.56)	114.50 (90.75-134.50)	0.166**
HDL	43.85 (35.97-50.25)	45.00 (39.00-53.00)	0.151**
Total cholesterol	183.10 (146.40-213.72)	186.50 (165.50-209.75)	0.399**
AIP	0.53 (0.34-0.75)	0.54 (0.36-0.69)	0.992**
CRI-I	3.39 (221-5.68)	3.48 (2.32-4.95)	0.988**
LDL-C/HDL-C Ratio	2.39 (1.89-3.08)	2.45 (2.03-2.97)	0.735**
AC	2.39 (1.21-4.68)	2.48 (1.32-3.95)	0.988**
PIV	301.88 (195.15-440.73)	248.91 (160.85-378.33)	0.026**
SII	535.51 (362.26-776.53)	486.55 (338.61-757.15)	0.694**

A total of 57.5% (n=84) had an AA diameter of 40-49 mm (Group 1), and 42.5% (n=62) had a diameter of 50 mm or more (Group 2). There was no significant difference between the calculated indices of Group 1, Group 2 and the control group (p>0.05) (Table 2).

**Table 2:** Comparison of indices in Group 1, Group 2 and the control group *Kruskal‒Wallis H test AIP: Atherogenic Index of Plasma; CRI-I: Castelli Risk Index-I; AC: Atherogenic Coefficient; PIV: Pan-Immune-Inflammation Value; SII: Systemic Immune-Inflammation Index

Indices	Patient Group (n=146)		Control Group (n=88)	p*
	Group 1 (AA 40-49 mm) (n=84)	Group 2 (AA ≥50 mm) (n=62)		
	Median (1-3 Quartiles)	Median (1-3 Quartiles)	Median (1-3 Quartiles)	
AIP	0.49 (0.31-0.75)	0.57 (0.35-0.77)	0.54 (0.36-0.69)	0.281
CRI-I	3.10 (2.05-5.62)	3.72 (2.28-5.89)	3.48 (2.32-4.95)	0.280
LDL-C/HDL-C Ratio	2.39 (1.90-3.15)	2.40 (1.86-3.02)	2.45 (2.03-2.97)	0.711
AC	2.10 (1.05-4.62)	2.72 (1.28-4.89)	2.48 (1.32-3.95)	0.280
PIV	308.26 (182.52-499.16)	295.89 (205.33-408.87)	248.91 (160.85-378.33)	0.077
SII	497.99 (338.49-776.74)	585.59 (365.66-778.31)	486.55 (338.61-757.15)	0.736

There was no significant correlation between the ascending aortic diameter and the AIP, CRI-I, LDL-C/HDL-C ratio, atherogenic coefficient, PIV, or SII index of patients with AAA (p>0.05) (Table 3).

**Table 3:** Relationships between the AA diameter and indices in the patient groups r: Spearman correlation coefficient AIP: Atherogenic Index of Plasma; CRI-I: Castelli Risk Index-I; AC: Atherogenic Coefficient; PIV: Pan-Immune-Inflammation Value; SII: Systemic Immune-Inflammation Index

Indices	Patient Group (n=146)	
	AA Diameter	
	r	p
AIP	0.087	0.299
CRI-I	0.087	0.297
LDL-C/HDL-C Ratio	-0.057	0.495
AC	0.087	0.297
PIV	-0.009	0.911
SII	0.138	0.096

**Figure 1:**
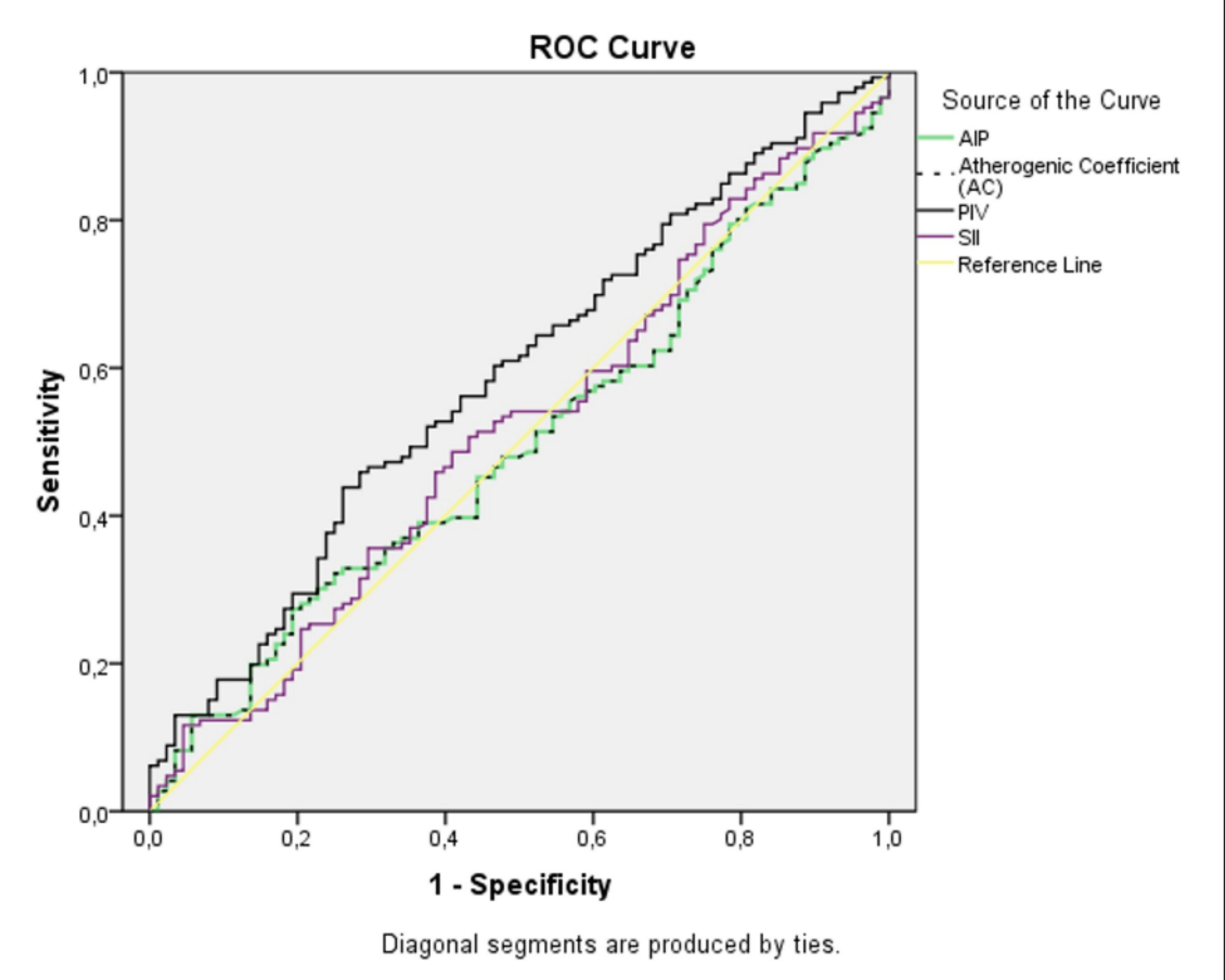
ROC curves for the calculated indices

The diagnostic decision-making properties of the indices determined in the study for predicting disease were examined with receiver operating characteristic (ROC) curves (Figure 1). The AUC values calculated from the ROC curves are shown in Table 4. ROC curve analysis revealed that the PIV index was significant (p=0.026).

**Table 4:** ROC analysis results of the calculated indices AIP: Atherogenic Index of Plasma; CRI-I: Castelli Risk Index-I; AC: Atherogenic Coefficient; PIV: Pan-Immune-Inflammation Value; SII: Systemic Immune-Inflammation Index; AUC: Area Under the Curve

Parameter	AUC (%95 GA)	p
AIP	0.500 (0.424-0.575)	0.992
CRI-I	0.499 (0.424-0.575)	0.988
LDL-C/HDL-C Ratio	0.487 (0.412-0.562)	0.735
AC	0.499 (0.424-0.575)	0.988
PIV	0.587 (0.513-0.662)	0.026
SII	0.515 (0.439-0.592)	0.694

## Discussion

The aim of this study was to investigate the predictive value of indices that indirectly indicate hyperlipidemia and inflammation in patients with AAA. This prospective study included 146 patients diagnosed with AAA and 88 controls. This study revealed that patients with AAA presented significantly higher PIV levels than controls did, suggesting that inflammation could be an important factor in AAA progression. However, there were no significant differences in the AIP, AC, or SII between the patient and control groups, nor was there a significant correlation between the aortic diameter and these indices. These findings suggest that while hyperlipidemia, as measured by the AIP and AC, may not play a direct role in AAA progression, inflammation indicated by PIV could be a crucial factor. This study highlights the potential utility of PIV as a predictive marker for AAA, especially in resource-limited settings.

Thoracic aortic aneurysms (TAA) rarely exhibit symptoms, with approximately 95% of patients being asymptomatic. These aneurysms can lead to serious complications, such as aortic dissection or rupture, and are often referred to as "silent killers." Roughly 22% of individuals succumb to these complications before reaching the hospital. Most aneurysms in the thoracic aorta occur in the root or ascending aorta, followed by the descending aorta, and they rarely occur in the arch [1,16].

Aortic aneurysms, particularly AAA, pose a significant health risk because of their potential for sudden rupture, leading to high mortality rates. Understanding the risk factors associated with aortic aneurysms is crucial for developing effective screening and prevention strategies. Numerous studies have identified various risk factors, including advanced age, male sex, smoking, hypertension, and inflammatory and specific genetic predispositions, as significant contributors to the development and progression of aortic aneurysms [3,17,18]. Recognizing and addressing these risk factors can aid in early detection and improve clinical outcomes for patients at risk. In our study, in line with the literature, male sex, advanced age, and hypertension were important risk factors in the AAA group.

Hyperlipidemia and inflammatory indices play important roles in the assessment and management of cardiovascular diseases. These indices are practical and valuable tools because of their ease of measurement, as they can be obtained from routine blood tests commonly performed in clinical settings. This makes them easily accessible and cost-effective. The ability to quickly obtain these indices allows for rapid risk assessment and stratification, which is necessary in both acute and chronic care settings. High levels of hyperlipidemia and elevated inflammatory indices are associated with an increased risk of cardiovascular events, making them important prognostic markers. They provide objective data that can guide clinical decision-making, helping identify high-risk patients who may benefit from targeted interventions and closer monitoring. Furthermore, understanding the interaction between lipid levels and systemic inflammation offers insights into the pathophysiology of cardiovascular diseases. This knowledge can inform personalized treatment strategies, such as the use of lipid-lowering therapies and anti-inflammatory agents, to mitigate risk and improve patient outcomes.

Atherogenic indices, including the AIP, are valuable tools for assessing clinical atherosclerotic diseases by taking into account lipoproteins and cardiovascular risk factors for refined evaluation and management. The AIP is calculated on the basis of the ratio of triglycerides to high-density lipoprotein cholesterol (TG/HDL-C). The AIP is a valuable tool for assessing cardiovascular diseases, particularly atherosclerosis. Studies have shown that the AIP is a strong predictor of coronary artery disease (CAD) risk and that higher AIP values are associated with an increased likelihood of CAD [19]. Additionally, the AIP has been found to be an independent risk factor for clinical atherosclerosis, demonstrating its importance in predicting and evaluating atherosclerotic conditions [20]. Moreover, research indicates that higher AIP values are linked to a greater plaque burden, highlighting its role in assessing the progression of atherosclerosis, especially in patients undergoing intravascular ultrasound [21]. Overall, the AIP is emerging as a promising and accessible index for risk assessment and monitoring of cardiovascular diseases, providing valuable insights into atherosclerotic conditions. The AIP and other indices offer better predictive value than individual lipids do. With this aim, we also evaluated several atherogenic indices. Our study revealed that the AIP and atherogenic coefficient were not significantly different between the AAA and control groups or between subgroups with different aortic diameters. These findings contradict those of several previous studies that identified elevated lipid levels as a risk factor for aortic aneurysms. The lack of a significant correlation between these lipid indices and aortic diameter in our study suggests that hyperlipidemia may not play a straightforward or direct role in the progression of aortic aneurysms. This discrepancy could be due to variations in the study populations, differences in the methods of lipid measurement, or other confounding factors not accounted for in this study.

Most importantly, patients with a history of atherosclerotic heart disease and cerebrovascular disease were excluded from our study. This suggests that, in the absence of risk factors like atherosclerosis, lipid parameters may not be useful as risk factors for aortic aneurysm.

Aortic aneurysms are serious vascular conditions closely associated with inflammation, and inflammatory processes play crucial roles in their pathogenesis and progression. Research has shown that inflammation-induced destruction of structural components within the arterial wall leads to the development of aortic aneurysms. In recent years, various biomarkers have been developed to systemically assess the inflammatory response. In this context, the PIV and SII have garnered attention as quantitative measures of the inflammatory burden and immune response. The SII combines basic hematological parameters such as neutrophil, lymphocyte, and platelet counts to provide an overall measure of inflammation. Conversely, the PIV is calculated via a combination of thrombocyte, lymphocyte, neutrophil, and platelet counts, offering a more specific indicator of systemic inflammation. The associations of these indices with cardiovascular diseases are highly important for risk stratification and patient prognosis. Studies in the literature indicate that high PIV and SII values correlate with an increased risk of cardiovascular events. There are different ways to detect inflammation, which is involved in the pathogenesis of many diseases. The relationship between inflammation detected by PIV and endothelial dysfunction is thought to be stronger than other inflammatory parameters such as SII. The Pan-immune-Inflammation Value (PIV) has been extensively studied in various cardiovascular contexts. Research has shown that high PIV levels are associated with an increased risk of contrast-induced nephropathy in patients undergoing coronary angiography for stable ischemic heart disease [22], predict severe coronary lesions in patients with non-ST-segment elevation myocardial infarction [23], and serve as significant predictors for vascular Behçet's disease [24]. Additionally, PIV has been linked to postcontrast acute kidney injury (PCAKI) in patients with acute coronary syndrome undergoing percutaneous coronary intervention, with higher PIV levels associated with a greater risk of developing PCAKI [14]. Collectively, these findings highlight the potential of PIV as a valuable biomarker for assessing cardiovascular conditions and complications, offering insights into risk prediction and disease severity evaluation. In our study, while the SII was not significantly different between the AAA group and the control group, PIV was significantly different. However, there were no significant differences in the two indices between the different subgroups in terms of aortic diameter. The AUC value of the PIV index was found to be 0.587 in the ROC curve analysis. This AUC value indicates that the level of accuracy of PIV in predicting the AAA presence is moderate. This finding indicates that PIV may be a potential auxiliary tool in the AAA risk assessment, but should not be used as a diagnostic tool alone. In addition, the threshold values used for the non-significant AUC values of other indices and the possible causes of this poor predictive performance are also discussed. These reasons include limited sample size, index sensitivity, and the multi-factor complex nature of AAA.

While our study found no significant correlation between aortic diameter and the indices AIP, AC, PIV, and SII, this may suggest that these markers are more indicative of systemic conditions rather than the specific pathological changes in the ascending aorta. The variability in patient characteristics likely contributed to this lack of association, underscoring the complexity of aortic aneurysm pathophysiology. Moreover, the variability in individual patient characteristics, such as differences in underlying comorbidities or genetic predispositions, may have diluted potential associations, leading to the observed results. This highlights the complexity of aortic aneurysm pathophysiology, where multiple factors likely interact in a multifaceted manner, rendering single biomarkers insufficient in capturing the entirety of the disease process. The study revealed statistically significant findings in areas such as gender distribution and chronic disease prevalence, but these do not necessarily translate to clinical significance, particularly given the small sample sizes and borderline p-values in some subgroups. The lack of significant differences in indices like AIP, AC, and SII, and their correlation with aortic diameter, may be due to the broader systemic nature of these markers or the heterogeneity of the study population. While the results offer valuable insights, they should be interpreted cautiously, considering the study's limitations. Future research with larger, more homogeneous cohorts is needed to validate these findings and explore additional biomarkers for assessing ascending aortic aneurysms.

The findings of this study highlight the novel role of the PIV as a potential marker in the management of AAA. The moderate accuracy of PIV, as indicated by an AUC of 0.587, suggests that while PIV alone may not suffice as a diagnostic tool, it could be valuable when integrated into broader screening protocols and patient monitoring strategies. This approach could enhance early detection and risk stratification, particularly in patients where traditional markers fall short.

Furthermore, this study opens up new avenues for future research. Investigating additional inflammatory markers or exploring the interplay between hyperlipidemia and inflammation in different patient populations could provide deeper insights into the complex pathophysiology of AAA. Expanding research to larger and more diverse cohorts would also be essential to validate these findings and potentially influence healthcare strategies and policy-making. The incorporation of PIV and similar indices into clinical practice could ultimately lead to improved patient outcomes by enabling more targeted and effective management of AAA.

Limitations

This study, while providing valuable insights into the potential role of inflammatory and lipid indices in the assessment of AAA, has several limitations that must be acknowledged. One of the primary limitations is the relatively small sample size, which may have impacted the statistical power of the study and could limit the generalizability of the findings. Another limitation is the potential for confounding factors that were not fully accounted for. While we attempted to control for known confounders through our study design and statistical analysis, it is possible that unmeasured variables, such as genetic predispositions, lifestyle factors, or undiagnosed comorbidities, may have influenced the results. These factors could affect the validity of our findings and should be explored in future research with more comprehensive data collection and adjustment techniques.

Additionally, the exclusion criteria, which were designed to eliminate patients with conditions that could interfere with the study's outcomes (e.g., recent major surgery, malignancy, severe renal or hepatic dysfunction), may have inadvertently narrowed the scope of the study. While necessary to maintain internal validity, these exclusions could limit the applicability of the findings to the broader population of AAA patients. In conclusion, while the results of this study offer promising insights, they should be interpreted with caution given these limitations. Future research with larger, more diverse cohorts and a broader range of measured variables will be essential to validate these findings and to further explore the complex interactions between the indices studied and the progression of AAA.

## Conclusions

Our results highlight the complexity of aortic aneurysm pathophysiology and suggest that inflammation, as indicated by PIV, may be a relevant factor, but hyperlipidemia may not simply contribute directly to the development and progression of AAA. We recommend that PIV be calculated at least once in patients diagnosed with AAA. Understanding the complex interactions of factors contributing to AAA will be crucial to developing effective risk stratification and management strategies for this potentially life-threatening condition. The fact that hyperlipidemia-related indices are not associated with AAA does not necessarily mean that it is not involved in etiopathogenesis. Larger studies are needed to demonstrate the association between AAA and hyperlipidemia.
